# Influence of Freezing and Storage Procedure on Human Urine Samples in NMR-Based Metabolomics

**DOI:** 10.3390/metabo3020243

**Published:** 2013-04-09

**Authors:** Manuela J. Rist, Claudia Muhle-Goll, Benjamin Görling, Achim Bub, Stefan Heissler, Bernhard Watzl, Burkhard Luy

**Affiliations:** 1 Department of Physiology and Biochemistry of Nutrition, Max Rubner-Institut (MRI), Haid-und-Neu-Str. 9, Karlsruhe 76131, Germany; E-Mails: Achim.Bub@mri.bund.de (A.B.); Bernhard.Watzl@mri.bund.de (B.W.); 2 Institute of Organic Chemistry, Karlsruhe Institute of Technology (KIT), Fritz-Haber-Weg 6, Karlsruhe 76131, Germany; E-Mails: Claudia.Muhle@kit.edu (C.M.-G.); Benjamin.Goerling@kit.edu (B.G.); Burkhard.Luy@kit.edu (B.L.); 3 Institute of Functional Interfaces, Karlsruhe Institute of Technology (KIT), Hermann-von-Helmholtz-Platz 1, Eggenstein-Leopoldshafen 76344, Germany; E-Mail: Stefan.Heissler@kit.edu; 4 Institute for Biological Interfaces, Karlsruhe Institute of Technology (KIT), Hermann-von-Helmholtz-Platz 1, Eggenstein-Leopoldshafen 76344, Germany; E-Mail: Burkhard.Luy@kit.edu

**Keywords:** NMR, metabolomics, urine, freezing, storage, SOP, pH, dry ice, CO_2_

## Abstract

It is consensus in the metabolomics community that standardized protocols should be followed for sample handling, storage and analysis, as it is of utmost importance to maintain constant measurement conditions to identify subtle biological differences. The aim of this work, therefore, was to systematically investigate the influence of freezing procedures and storage temperatures and their effect on NMR spectra as a potentially disturbing aspect for NMR-based metabolomics studies. Urine samples were collected from two healthy volunteers, centrifuged and divided into aliquots. Urine aliquots were frozen either at −20 °C, on dry ice, at −80 °C or in liquid nitrogen and then stored at −20 °C, −80 °C or in liquid nitrogen vapor phase for 1–5 weeks before NMR analysis. Results show spectral changes depending on the freezing procedure, with samples frozen on dry ice showing the largest deviations. The effect was found to be based on pH differences, which were caused by variations in CO_2_ concentrations introduced by the freezing procedure. Thus, we recommend that urine samples should be frozen at −20 °C and transferred to lower storage temperatures within one week and that freezing procedures should be part of the publication protocol.

## 1. Introduction

The general aim of metabolomics studies is the identification of mostly subtle differences in biological systems under defined conditions. The detection of those subtle differences usually requires the maximum possible reproducibility of both sample preparation and measurement. The smallest changes in the sample treatment or analysis can confound the desired effects and lead to noise in corresponding data that adds to the underlying biological variations and complicates data interpretation. It is therefore necessary to study in detail every single step in a standard operating procedure (SOP) and eliminate potential disturbances. Practically all studies on biological systems involve freezing and storage steps, and freezing conditions may vary—even within a study—usually because of local conditions, like the accessibility or inaccessibility of a freezer in close proximity to the site where the samples are generated and the corresponding use of dry ice for freezing or intermediate storage at a certain floor, *etc.* Therefore, our aim was the detailed investigation of common freezing conditions and their effects on resulting NMR spectra using buffered urine as one of the most commonly studied biological matrices.

NMR is widely used as an analytical technique in the field of metabolomics [1,2,3]. Often, it is applied as a non-targeted fingerprinting method, *i.e.*, only the pattern of the metabolite spectra is analyzed rather than the identity and quantity of underlying compounds [1]. In these cases, the peak positions in the spectra are just as important as their intensities. For most detectable metabolites, this is not an issue, because their signals are not influenced by variations in the samples (e.g., hippurate), but roughly 10%–20% of the compounds are sensitive towards pH or salt concentration changes (e.g., citrate, histidine (His) and its derivates [4,5]), which results in peak shifts. In this respect, urine is difficult to handle, as it shows large differences in pH and salt concentration between individuals, which is the reason why it is common practice to try to adjust the pH by adding a highly concentrated buffer [5,6,7]. Still, there are large inter-individual differences in urine composition, and even with a final concentration of 150 mM phosphate buffer, the pH of the resulting urine samples varies—in our experience, approximately between pH 6.8 and 7.2. Different strategies to account for these sample differences are applied, for example, using higher buffer concentrations [8] or adjusting the pH [9], but it is obvious that no further variations should be introduced by sample handling, storage or preparation.

Although some basic studies have been published that investigated the influence of sample treatment and storage temperature on NMR-based urine metabolite profiles in the context of the interpretation of NMR-based urine metabolomics studies [8,10,11,12], the number of publications is limited, and some questions remain open.

Based on principal component analysis (PCA) of binned NMR spectra, some authors have reported that there is no difference between samples stored at different temperatures for several months, as long as the samples are cooled to 4 °C or frozen at ≤ −20 °C [8,10]. Saude and Sykes looked at changes of a set of 55 predefined metabolites in urine samples that were pre-treated in different ways and stored at different temperatures for four weeks. They found no significant changes in metabolite concentrations over time when samples conserved with NaN_3_ were stored at −80 °C and only little changes in samples stored at 4 °C [11]. For these studies, it can only be speculated that urine samples were also frozen at the temperatures at which they were stored, since this information was not given. In fact, most publications in the field of metabolomics note only the storage temperature of the samples prior to analysis, but for practical reasons, the actual freezing procedure may require conditions that are different from long-term storage conditions. This influence of the freezing procedure on NMR spectra of urine samples has not been reported as such in the literature. Recently, Bernini *et al.* observed differences in urine NMR spectra depending on sample processing (centrifugation or not) and freezing/storage temperature [5]. In samples that were relatively slowly frozen and stored at −80 °C using a freezer, the difference between pre-centrifuged and non-pre-centrifuged samples was largest, while rapid freezing and storing samples in liquid nitrogen (lN_2_) resulted in only small differences between samples that were centrifuged or not [5]. Thus, snap freezing samples may be a useful solution, not only when working with tissues or cells, but also with biofluids that may contain some cells. In many laboratories, rapidly freezing samples can more easily be achieved with dry ice, which is solid carbon dioxide (CO_2_), but the influence of freezing urine on dry ice on the respective urine NMR spectra has never been reported.

Therefore, the aim of this work was to test different combinations of practically relevant freezing procedures, including dry ice and storage temperatures of human urine samples, and to investigate their effect on NMR spectra in order to develop a SOP that is practicable in our hands and could be useful also for other scientists using NMR-based metabolomics on urine samples.

## 2. Results and Discussion

**Scheme 1:**
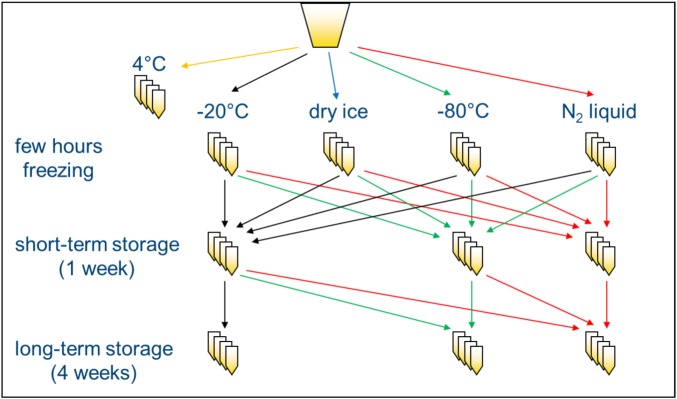
Study design: two urine samples were each divided into 99 aliquots and frozen (or not) at different temperatures/conditions. Thereafter, samples were either left at the same temperature for short- or long-term storage or moved to a different short- or long-term storage temperature, as shown by the arrows. N = 3 for each condition.

In order to investigate the influence of freezing and storage conditions on NMR spectra of human urine, a systematic screening was performed. Urine samples of two volunteers were centrifuged to eliminate bacteria or other cellular components [5], and supernatants were frozen at −20 °C, on dry ice (−78.5 °C), at −80 °C or in lN_2_ (−196 °C) and either analyzed after a few hours (“freezing”) or stored at the same or a different temperature for one week before NMR measurement (“short-term storage”) or stored at the same or a different temperature for an additional four weeks (“long-term storage”) and analyzed thereafter (Scheme 1). Samples that were not frozen, but kept at 4 °C for the same time as needed for freezing the samples, were used as a reference. Subsequently, standardized 1D ^1^H-NOESY NMR spectra with presaturation were acquired and processed uniformly for all samples, as described in the Experimental Section. In addition, several control experiments were performed to corroborate and extend the results described in the following subsections.

### 2.1. Influence of Freezing and Storage Conditions on NMR Spectra of Urine Samples

**Figure 1:**
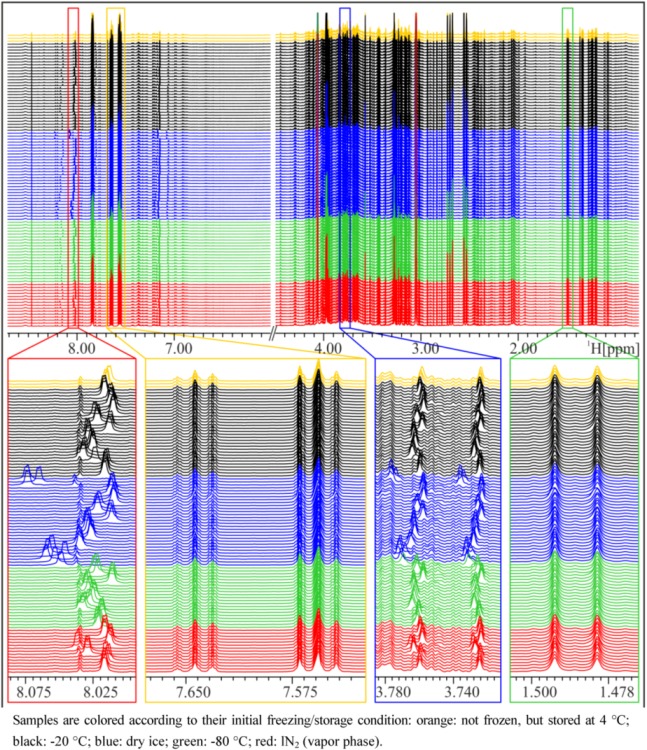
NMR spectra of a spot urine sample of a female subject, frozen and stored under different conditions. Upper panel: overview of whole NMR spectra—urea and water region excluded—demonstrating overall good reproducibility. Lower panels: zoom into relatively constant regions and variable regions with shifted peaks.

**Figure 2:**
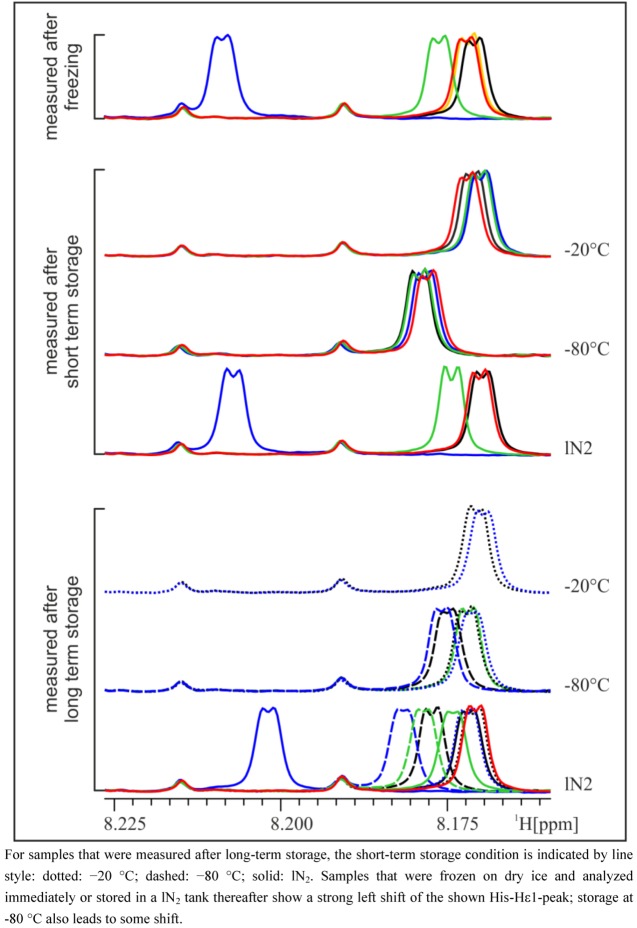
Zoom into pH-sensitive region of NMR spectra of the 24 h urine of a male subject. Samples are colored according to their initial freezing/storage condition (for the legend, see Figure 1) and grouped according to their last storage condition.

In general, NMR spectra of different aliquots of the same subject prepared independently at several occasions within five weeks are, as expected, very similar and again demonstrate the impressive reproducibility of NMR spectroscopy (Figure 1). However, closer inspection of the NMR spectra reveals that there are differences depending on the freezing procedure (Figure 1). Particularly in the aromatic region, marked peak shifts can be observed after freezing samples on dry ice or in a −80 °C freezer, the most prominent being the shift of the Hε1 peak of His at approximately 8.0 to 8.2 ppm, which shifts by about 0.04 to 0.06 ppm after freezing on dry ice, depending on the volunteer. Other peaks that show variations depending on the freezing procedure include the Hδ2 peak of His (7.1–7.2 ppm) and the corresponding peaks in His derivatives (1- or 3-methyl histidine, His dipeptides), but also creatinine (4.06 ppm) or citrate (2.7 ppm). These signals are known to be sensitive to pH changes [4,5]. The observed differences weakened or disappeared after a longer storage time, depending on the type of long-term storage (Figure 2). Whereas after storage of one or five weeks at −20 °C, the shifted His-Hε1-peak moved back to the same position as in the reference samples (not frozen) or samples that were frozen and stored at −20 °C, storage in lN_2_ conserved the state of the sample frozen in any condition over the five-week storage period. Interestingly, storage of urine samples at −80 °C introduced some unexpected variability in NMR spectra, and generally, variability between replicate NMR spectra was largest in samples frozen on dry ice. Remarkably, under all conditions where differences were observed, only the peak positions change, but not the intensity of the peaks, which means that there are no concentration changes of metabolites. In other words, the observed chemical shift variations due to different freezing or storage conditions are not caused by the decomposition of macromolecules or degradation of metabolites. Therefore, it can be assumed that the observed chemical shift changes can affect pattern recognition/fingerprinting analyses of resulting NMR spectra, but not a targeted quantitative evaluation of NMR data.

**Figure 3:**
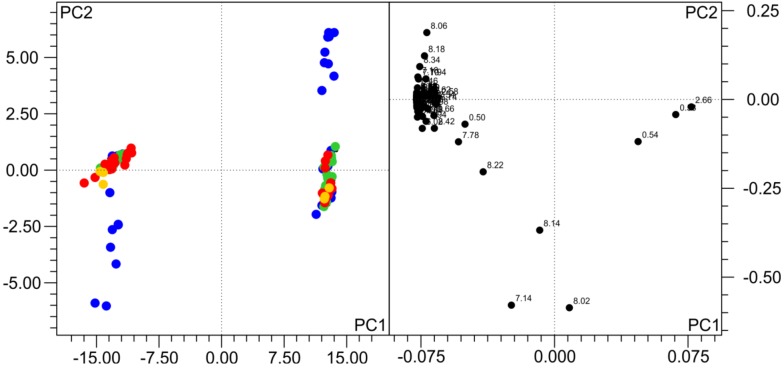
Principal component analysis (PCA) of 196 samples from the two volunteers frozen and stored under different conditions. Samples colored according to freezing temperature (for the legend, see Figure 1). Subjects are separated in the scores plot (left), but, also, samples frozen on dry ice are distinct from others. The loadings plot (right) shows the variables (bins) responsible for the separation. In the first two PCs > 96% of the variance in the data are explained. R2 = 0.976, Q2 = 0.959.

To get an overview of the general variability of NMR spectra and in order to test if the observed changes indeed influence pattern recognition analyses, we have performed a PCA analysis. In a PCA of a single subject, only samples that were frozen on dry ice and either analyzed immediately or stored thereafter in lN_2_ are distinct from the others (not shown). When all the samples of the two subjects are analyzed together, as also observed by others [8,10,12], the inter-individual differences between urine samples are by far larger than the influence of the freezing procedure, but still, the samples frozen on dry ice and analyzed directly or stored in lN_2_ thereafter are distinct from all others (Figure 3). The bins driving this separation mainly belong to His and its derivates, which are known to be pH-sensitive.

Therefore, in most cases, the result or interpretation of a metabolomics experiment will in practice not be affected by the freezing condition or storage temperature, with one exception: when freezing the fresh samples on dry ice, the changes in NMR spectra are so large that they can affect the PCA analysis.

**Figure 4:**
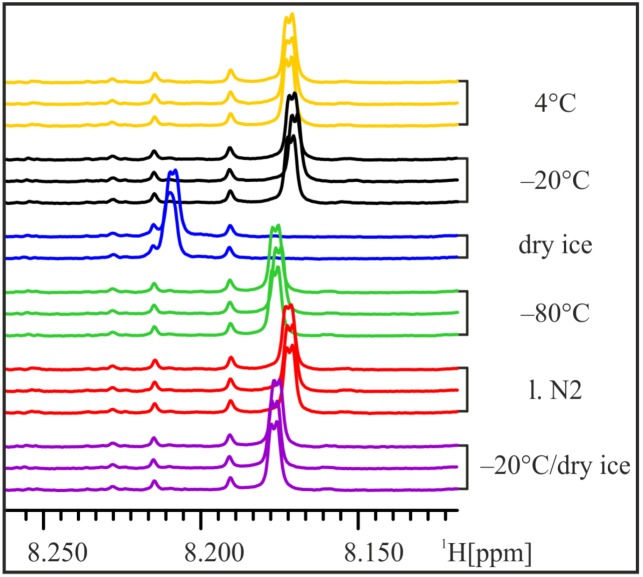
Transport on dry ice. In order to simulate transport of frozen samples on dry ice, aliquots of the male 24 h urine sample were frozen at −20 °C for 15 h and then stored on dry ice for 1.5 h before NMR sample preparation. The position of the His-Hε1-peak still shifts slightly, but not more than in samples frozen at −80 °C.

In order to mimic the (in-house) transport of frozen samples on dry ice, three aliquots of one urine sample were frozen at −20 °C for 15 h and then stored on dry ice for 1.5 h before thawing and NMR analysis. The NMR spectra of these “transport” samples do not show the strong shift of the His-Hε1-peak and strongly resemble samples frozen at −80 °C (Figure 4). Thus, we conclude that already frozen samples are less influenced by subsequent storage on dry ice and transport of frozen samples on dry ice at least for short time periods seems to have little impact on urine samples.

### 2.2. Influence of Freezing and Storage Conditions on pH of Urine Samples

**Figure 5:**
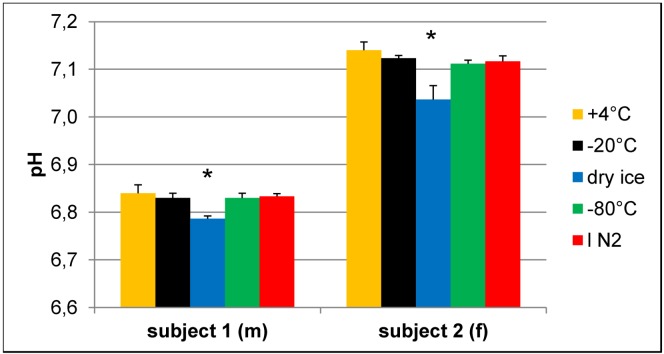
Effect of freezing on pH of urine samples.pH values were determined in buffered urine samples with a final concentration of 150 mM phosphate buffer after NMR experiments. Samples that were frozen on dry ice have a significantly different pH compared to all other samples of the same volunteer. N = 3, values given as the mean + SD; * *p* < 0.05 *vs.* all other samples of the same subject (one-way ANOVA).

The aromatic proton peaks of His are known to be sensitive to changes in pH [4,5], and imidazole is used as a pH reference in NMR-based metabolomics [13]. Therefore, the observed differences in peak positions in NMR spectra (Figure 1) seem to be mainly based on pH variations that are introduced during the freezing and (short-term) storage conditions. To confirm this hypothesis, the pH was measured in some of the samples after the NMR experiment, *i.e.*, with buffer added (Figure 5). Although the changes in pH are small, due to the added buffer, there are reproducible pH changes of about 0.05 to 0.1 pH units in the samples that were frozen on dry ice and analyzed immediately or stored thereafter in lN_2_. Thus, the recorded pH differences cor2relate with the peak shifts observed in the NMR spectra.

### 2.3. Changes in NMR Spectra and pH Depend on Storage Vial

As we observed pH changes that perfectly correlate with measured chemical shift changes, we performed further experiments to determine the underlying cause of the observed variations. A maximum change in pH from 7.2 to 7.1 was observed predominantly for samples stored on dry ice, which is solid CO_2_. CO_2_ partially reacts with water to form carbonic acid or hydrogen carbonate, its respective anion. It is thus highly likely that the observed pH change was caused by dissolution of CO_2_ rather than other changes caused by the low temperatures, such as sample composition or degradation.

**Figure 6:**
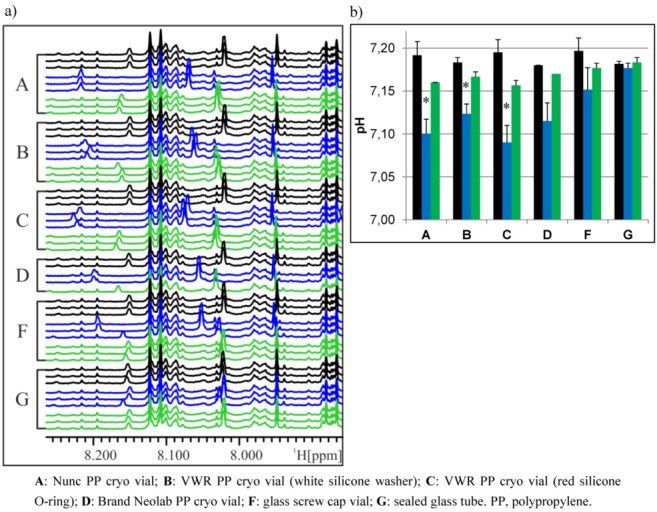
Influence of storage vial on NMR spectra and pH of urine samples frozen under different conditions. (**a**) Variable region of NMR spectra of urine samples frozen at different temperatures and in different PP cryo or glass vials, colored according to freezing condition. Black: −20 °C; blue: dry ice; green: −80 °C. (**b**) pH values of corresponding urine samples, colored according to freezing temperature (see above). N = 3, except D (n = 1–2), values given as the mean + SD. * *p* < 0.05 *vs.* all other samples of the same vial (two-way ANOVA) (there are no statistics for D, due to a low number of replicates).

Since polypropylene (PP) is permeable for many gases, including CO_2_, and since we could think of no other source of acidification of the samples, we tried to test the hypothesis that CO_2_ permeation of PP vials is the cause for the observed pH change in the urine samples analyzed. Therefore, we used a third urine sample that was aliquoted into different PP cryo vials, screw cap glass vials or glass tubes, which were sealed gas-tight. Samples in each type of vial were frozen at −20 °C, on dry ice or at ‑80 °C for approx. 16 h to 2.5 days before sample preparation. NMR analysis of these urine samples showed again the previously observed peak shifts of the samples frozen in cryo vials on dry ice, but only small changes in NMR spectra of samples frozen in glass screw cap vials and practically no peak shifts in samples frozen in sealed glass tubes (Figure 6a). At the same time, the pH differences between the samples stored at different conditions were weaker in screw cap glass vials and absent in sealed glass tubes (Figure 6b). As observed in previous experiments, the reproducibility of NMR spectra is generally slightly less in samples frozen in a ‑80 °C freezer compared to ‑20 °C and even less in samples frozen on dry ice. The variability observed with samples stored in glass vials may result from non-uniform tightening of the screw cap or not a 100% gas-tight sealing of glass tubes.

### 2.4. Changes in pH Are Caused by CO_2_

To prove whether storage under the above mentioned conditions leads to a measurable amount of CO_2_ or hydrogen carbonate in the solution, we employed infrared spectroscopy. Since most compounds of urine give rise to infrared absorption bands, which could impede the identification of the hydrogen carbonate band in the spectrum due to their high concentration, for this experiment, we used buffer samples instead. One point two milliliter cryo vials containing 1 mL of 10% NMR buffer, corresponding to the same final concentration as in prepared urine samples, were frozen overnight either at −20 °C, on dry ice or at −80 °C. A cryo vial with buffer with the original pH of 7.2 that was stored at 4 °C served as reference sample and was subtracted during the measurement. The pH of the solution was measured immediately after FTIR spectroscopy, which revealed that the sample stored on dry ice had undergone a pH change to 6.9, while the pH of the other two remained unchanged.

**Figure 7:**
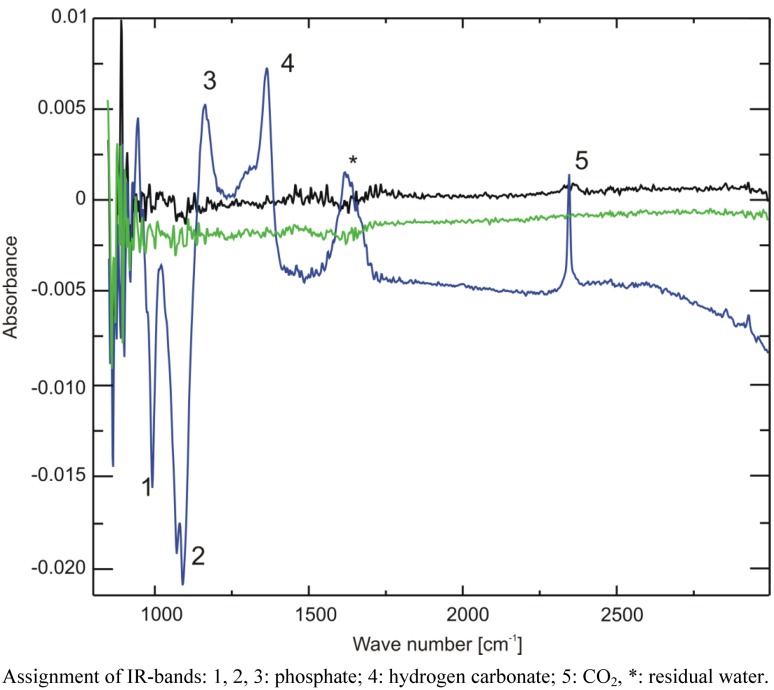
Attenuated Total Reflection Fourier-Transform Infrared Spectroscopy (ATR-FTIR) spectra of three buffer samples frozen overnight at −20 °C (black), −80 °C (green) or on dry ice (blue). A spectrum of buffer stored overnight at 4 °C was used as a reference and was subtracted from all other spectra.

The IR spectra are displayed in Figure 7. While the two samples stored at −20 °C and −80 °C show no difference to the reference sample (the spectrum is devoid of a signal, due to ideal subtraction of water and buffer absorption bands), both positive (increase) and negative (decrease) changes of absorption can be seen in the region between 900 and 2,500 cm^−1^ for the sample stored on dry ice. The phosphate bands at 1,080 and 990 cm^−1^ decrease, giving rise to negative intensity, while the one at 1,160 cm^−1^ increases, all corresponding with a lower pH [14]. Furthermore, a positive band at 1,361 cm^−1^ can be discerned. Through comparison with a sample of 40 mM sodium hydrogen carbonate dissolved in the same buffer, this band could be identified as hydrogen carbonate, corresponding with an increased hydrogen carbonate concentration in the buffer. This proves—together with the positive band at 2,343 cm^−1^ corresponding to CO_2_—that indeed CO_2_ is the cause of the observed pH changes.

Is it possible to estimate how much CO_2_ is dissolved in the samples? In the IR spectra, the phosphate band at 1,160 cm^−1^ partially overlaps with the hydrogen carbonate band, and it is extremely sensitive to pH changes in the range of 10^−2^ around its pK_A_ value. This renders a precise integration of the hydrogen carbonate band difficult. Instead, we calculated a lower value for the amount of dissolved hydrogen carbonate from the observed pH change and the known buffer concentration using the Henderson-Hasselbalch Equation (1):

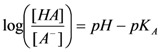
(1)


The pK_A_ of phosphate is 7.21. At pH 7.2, the ratio between H_2_PO_4_^−^ and HPO_4_^2−^ is approximately 1, at pH of 6.9 it is approximately 2. With a total buffer concentration of 150 mM (H_2_PO_4_^−^ + HPO_4_^2−^), we calculate 75 mM for each species at pH 7.2. At pH 6.9, the concentrations are 100 mM for H_2_PO_4_^−^ and 50mM HPO_4_^2−^. Thus, to shift the ratio from 1 to 2 in our samples, 25 mM H_3_O^+^-ions are necessary. They must come from the dissolution of CO_2_ and subsequent dissociation of the carbonic acid. Since carbonic acid is a weak acid, not all carbonic acid is dissociated, and we estimate that 25 mM concentration is just a lower estimate for the amount of dissolved CO_2_.

The diffusion of CO_2_ into cryo vials and hydrogen carbonate formation in aqueous solutions with concomitant changes in pH is detected in NMR analyses, due to the simple sample preparation that preserves as much as possible of the original properties of the samples. Other techniques that are frequently used in metabolomics, such as mass spectrometry and gas or liquid chromatography, require more complicated sample preparation and include extraction with organic solvents, purification by solid phase extraction or derivatization of samples. The ionization behavior of samples, however, certainly depends on the pH or charge state of corresponding molecules. How far such techniques may be affected by pH differences in the raw samples will strongly depend on sample treatment and cannot be judged by the presented data.

## 3. Experimental Section

### 3.1. Materials

KH_2_PO_4_ and KOH were from Merck (Darmstadt, Germany); trimethylsilyl propionic adic-d4 (TSP, 98 atom% D), NaN_3_ and D_2_O (99.9 atom% D) were from Aldrich (Steinheim, Germany). All chemicals were analytical grade and used without further purification.

Different 1.2 mL PP cryo vials with an internal thread were obtained from VWR (Bruchsal, Germany), Nunc or Brand (Neolab, Heidelberg, Germany).

NMR tubes were from Wilmad (Rototec-Spintec GmbH, Griesheim, Germany).

### 3.2. Volunteers, Sample Collection, Freezing and Storage

Spot or 24 h urine samples were collected from two healthy volunteers (one female, one male), centrifuged at 3,000 rpm (~1,850 rcf) for 10 min at 4 °C in a Jouan CR 422 centrifuge to eliminate bacteria or other cellular components that could disintegrate during freezing [5] and aliquoted into PP cryo vials (VWR). Urine aliquots were frozen for several hours, either at −20 °C, on dry ice, at −80 °C or in lN_2_, and then either analyzed by NMR directly or stored at −20 °C, −80 °C or in the lN_2_ vapor phase for 1–5 weeks (Scheme 1). An additional 24 h urine sample collected from a third volunteer (female) was centrifuged as described above and aliquoted into four different types of PP cryo vials, screw cap glass vials or glass tubes that were sealed gas-tight. The samples in the different vials were then frozen and stored overnight either at −20 °C, on dry ice or at −80 °C. Volunteers were part of a larger study that is being conducted at the Max Rubner-Institut in Karlsruhe. The study protocol was approved by the Ethical Committee of the State Medical Chamber Baden-Württemberg, Germany (F-2011-051). All participants gave written consent prior to the study.

### 3.3. Sample Preparation and NMR Analysis

For NMR analysis, thawed urine samples were mixed by inverting them 8–10 times, centrifuged at 4,000 rpm (~3,300 rcf) for 10 min at 20 °C in a Jouan CR 422 centrifuge to remove any non-soluble particles and 540 µL of the supernatants were mixed with 60 µL NMR buffer (1.5 M KH_2_PO_4_, 2 mM NaN_3_, 0.1% TSP in D_2_O, pH 7.1), transferred to a 5 mm NMR tube (Wilmad) and measured in a Bruker Avance III 600 MHz NMR spectrometer equipped with a TCI inversely detected cryoprobe (Bruker BioSpin GmbH, Rheinstetten, Germany) at 300 K (27.1 °C) using a 1D NOESY experiment with presaturation for water suppression. A prescan delay of 4 s was used together with a mixing time of 10 ms. Pulse lengths were determined automatically by the Bruker AU program, pulsecal. 64k complex data points corresponding to a sweep width of 12,345.6 Hz were recorded. All spectra were treated identically using an exponential apodization function, introducing an additional linewidth of 0.3 Hz and automated phasing, baseline correction and referencing using the Bruker macro, apk0.noe. Each sample was analyzed in triplicate using independently prepared samples.

### 3.4. Sample Preparation and Attenuated Total Reflection Fourier-Transform Infrared Spectroscopy (ATR-FTIR)

Cryo vials containing 1 mL diluted (10%) NMR buffer, pH 7.2, were frozen and stored overnight on dry ice, at −20 °C or at −80 °C, respectively (2 samples per storage condition). A buffer sample stored overnight at 4 °C served as the reference.

A Bruker Tensor 27 Fourier Transform IR spectrometer (Bruker Optik GmbH, Ettlingen, Germany), with a Bruker Bio ATR 2^®^ cell was employed to obtain the IR spectra of these samples. The special design of the cell provides constant temperature control of the sample solutions. The spectra were measured at 25 °C. The vials were inverted several times before the solution was applied to the measurement cell. Samples were allowed to temperature equilibrate for 2 minutes prior to measurement.

The used ATR cell was equipped with an ATR crystal made from silicon with nine reflections, 64 scans in the 4,000–850 cm^−1^ spectral range were recorded on a photovoltaic MCT-detector with a scanner velocity of 20 kHz and a spectral resolution of 4 cm^−1^. The reference spectra were taken with the buffer solution stored at 4 °C before measurement. For data acquisition and evaluation, the Bruker OPUS^®^ software, version 5.5, was used.

### 3.5. pH Measurements

In some urine samples, pH was measured after the NMR experiments using a Schott portable pH-meter with an InLab^®^ Expert Pro microelectrode. After the ATR-FTIR measurement, the pH of all buffer samples was measured with the same instrument that was calibrated with three reference solutions prior to the measurement.

### 3.6. Data Analysis

NMR spectra were displayed and analyzed using AMIX 3.9.10 (Bruker BioSpin GmbH, Rheinstetten, Germany). For the PCA, 196 NMR spectra were binned to 0.04 ppm in the region of 0.5–9 ppm, with the water and urea peaks excluded (6.0–4.5 ppm), yielding 177 bins, un-normalized bins were scaled to unit variance and the first 4 PCs were calculated, with PC1 and PC2 explaining > 96% of variance.

For the statistical tests on pH values of urine samples, a one-way and two-way ANOVA was performed, respectively, using SigmaPlot 11.0 (Systat Software GmbH, Erkrath, Germany).

## 4. Conclusions

In metabolomics studies, often subtle changes need to be detected in order to find physiologically relevant differences between study groups. Every step along the way, from sample collection to data analysis, needs to be carefully controlled, and there are many efforts to standardize protocols and reporting them, e.g., for sample collection and storage [15]. The actual freezing procedure, however, has not been addressed so far, and indeed, in most publications in the field of metabolomics, the freezing condition of samples is not mentioned. However, although for practical reasons, the actual freezing procedure of collected samples may involve conditions (e.g., temperature) that are different from long-term storage, there are only a few examples in the literature where freezing conditions are given [16,17,18]. In this work, we have systematically screened different freezing conditions and storage temperatures of human urine samples and investigated their effects on the metabolic fingerprint of samples analyzed by NMR.

Although the overall reproducibility of NMR is remarkable over the more than five weeks studied, we have observed differences in certain areas of the NMR spectra that indicate that some metabolites are affected by the freezing conditions. In some cases (for example His and derivates), this effect was large enough to potentially affect the results of a metabolomics study based on PCA. While freezing samples on dry ice showed the strongest effect, freezing and/or storage at −80 °C also induced changes in NMR spectra. These changes were found to be caused by changes in pH (acidification) that are, in turn, caused by an increase in CO_2_ and hydrogen carbonate in the sample, due to diffusion of CO_2_ through PP storage vials. In contrast, samples frozen in glass screw cap vials or sealed glass tubes did not show similar changes. The diffusion of CO_2_ into PP cryo vials while freezing samples on dry ice can easily be explained, since dry ice is solid CO_2_ and PP is known to be permeable for CO_2_. Using FTIR spectroscopy, we have shown an increase in CO_2_ and hydrogen carbonate concentration by at least 25 mM in buffer samples frozen on dry ice. Besides the effect for dry ice, we also have observed small, but unambiguously measurable, chemical shift changes for urine samples frozen or stored at −80 °C, affecting the same pH-dependent NMR signals. However, the effect seems to be variable and is not as well reproducible as the effects seen for dry ice. We can only speculate about the cause of the latter observation: the freezing temperature of CO_2_ is −78.5 °C, so it can be assumed that some CO_2_ from the air will solidify in a −80 °C freezer, eventually leading to some amount of dry ice. Depending on the intensity of use of the freezer and the inside temperature, which typically varies between −76 °C and −82 °C, it could be possible that the atmosphere inside a −80 °C freezer is at times enriched with CO_2_ compared to the normal room air or to a −20 °C freezer. This could also explain the here observed variability of NMR spectra of samples frozen or stored at −80 °C, which was always slightly larger than in samples frozen or stored at −20 °C or in lN_2_. Unfortunately, we were not able to measure directly the CO_2_ concentrations inside our −80 °C freezer, and we did not see changes in CO_2_ or hydrogen carbonate concentrations in the few buffer samples frozen at −80 °C. We, therefore, cannot fully exclude that also other mechanisms besides the pH change due to an increased CO_2_-level may contribute to the variability in urine samples frozen and stored at −80 °C. In all cases, the sources of variability described by Bernini *et al.* [5] are not likely to contribute to the observed variations, since their protocol of pre-centrifuging urine samples before freezing was followed to avoid breaking of cells and since chemical reactions, bacterial growth or enzymatic reactions should take place in all samples alike and have been described to lead to an alkalinization of urine samples in contrast to the acidification observed here.

Although it may be argued that urine from 24 h collections are usually frozen directly in the long-term storage device, local conditions or study design—such as in our own study center—still might make it necessary to use a different freezing procedure for short-term intermediate storage with the very same preconditions as described here.

Also, we have investigated urine from only two persons. This, however, is only a problem as long as statistic features are studied. In the present study, instead, we give a defined cause to the observed changes in spectra and consolidate the findings with further samples and with three different types of measurements, which all lead to the same coherent conclusion that CO_2_ is the cause of the observed pH changes.

The changes observed in this study may not be a problem in targeted/quantitative analyses of NMR spectra, where peaks are annotated to a metabolite and integrated for quantification, and the exact peak position is not as relevant. On the other hand, the described changes can easily be avoided by carefully choosing the freezing and storage conditions. Based on the results of this study, it seems that dry ice cannot be recommended to freeze samples, unless all samples are treated in the same way, but dry ice should have little effect during short transport periods of already frozen samples.

Thus, we would recommend that, if freezing samples in lN_2_ is not feasible, urine samples should be frozen at −20 °C and transferred to lower storage temperatures (−80 °C, lN_2_) within one week. If this is not possible, all samples of a study should be frozen in the same way. Finally, independent of the protocol applied, the freezing procedure should be part of every metabolomics publication.
